# Trends and predictors of appropriate complementary feeding practices in Nepal: An analysis of national household survey data collected between 2001 and 2014

**DOI:** 10.1111/mcn.12564

**Published:** 2017-11-17

**Authors:** Muzi Na, Víctor M. Aguayo, Mary Arimond, Pradiumna Dahal, Bikash Lamichhane, Rajkumar Pokharel, Stanley Chitekwe, Christine P. Stewart

**Affiliations:** ^1^ Program in International and Community Nutrition, Department of Nutrition University of California Davis CA USA; ^2^ Department of Nutritional Sciences, College of Health and Human Development The Pennsylvania State University, University Park PA USA; ^3^ Nutrition Section, Programme Division United Nations Children's Fund (UNICEF) New York NY USA; ^4^ Center for Dietary Intake Assessment FHI 360 Washington DC USA; ^5^ United Nations Children's Fund (UNICEF) Regional Office for South Asia Kathmandu Nepal; ^6^ Child Health Division Government of Nepal Ministry of Health Department of Health Services Kathmandu Nepal

**Keywords:** complementary feeding, DHS, MICS, multilevel models, Nepal, trends

## Abstract

There is evidence that suboptimal complementary feeding contributes to poor child growth. However, little is known about time trends and determinants of complementary feeding in Nepal, where the prevalence of child undernutrition remains unacceptably high. The objective of the study was to examine the trends and predictors of suboptimal complementary feeding in Nepali children aged 6–23 months using nationally representative data collected from 2001 to 2014. Data from the 2001, 2006, and 2011 Nepal Demographic and Health Surveys and the 2014 Multiple Indicator Cluster Survey were used to estimate the prevalence, trends and predictors of four WHO‐UNICEF complementary feeding indicators: timely introduction of complementary foods (INTRO), minimum meal frequency (MMF), minimum dietary diversity (MDD), and minimum acceptable diet (MAD). We used multilevel logistic regression models to identify independent factors associated with these indicators at the individual, household and community levels. In 2014, the weighted proportion of children meeting INTRO, MMF, MDD, and MAD criteria were 72%, 82%, 36% and 35%, respectively, with modest average annual rate of increase ranging from 1% to 2%. Increasing child age, maternal education, antenatal visits, and community‐level access to health care services independently predicted increasing odds of achieving MMF, MDD, and MAD. Practices also varied by ecological zone and sociocultural group. Complementary feeding practices in Nepal have improved slowly in the past 15 years. Inequities in the risk of inappropriate complementary feeding are evident, calling for programme design and implementation to address poor feeding and malnutrition among the most vulnerable Nepali children.

## 1 INTRODUCTION

South Asia has achieved significant progress in reducing child undernutrition, with a decline in the prevalence of stunting in children under five from 56% to 36% in the past 2 decades (Stevens et al., 2012). However, the rate of decline in recent years is not on track to meet the Sustainable Development Goal target of a 40% reduction in stunting by 2025 (United Nations, 2015). South Asia remains a “hot‐spot” in terms of the burden of child undernutrition: Four in 10 stunted children worldwide live in South Asia, and the total number of affected children is about 65 million (UNICEF, 2015).

In 2014, 37% of children under five are stunted in Nepal (Central Bureau of Statistics, 2015), and the prevalence is comparable to the regional average. What is more worrisome, however, is the slowdown in the rate of reduction of child stunting from an average annual rate of decrease of 3.4% per year between 2001 and 2011 to 2.6% per year between 2011 and 2014. In addition, the proportion of wasted children has stagnated at 11.2–11.3% between 2001 and 2014, and Nepal currently ranks 111th out of 130 countries globally on nutrition indicators (International Food Policy Research Institute, 2016).

Among all the causes of stunted growth in children, inadequate child feeding is one of the most proximal and immediate determinants (Black et al., 2013; Stewart, Iannotti, Dewey, Michaelsen, & Onyango, 2013). Suboptimal child feeding, defined by the World Health Organization (World Health Organization, 2010), includes delayed initiation of breastfeeding, shorter duration (<6 months) of exclusive breastfeeding, discontinuation of breastfeeding before 2 years of age, delayed introduction of complementary foods, and inadequate quantity and quality of complementary foods given to children aged 6–23 months. In Nepal, children are predominantly breastfed in the first 6 months of life (75%) and are likely to continue to breastfeed until 2 years of age (87%), although the proportion of children who initiate breastfeeding within 1 hr of birth (49%) and are exclusively breastfed in the first 6 months of life (57%) is lower (Central Bureau of Statistics, 2015). Compared to the breastfeeding indicators, complementary feeding indicators in children aged 6–23 months reflect much poorer practices: It is estimated that only one in three Nepali children is fed with the minimum frequency and dietary diversity (Central Bureau of Statistics, 2015). It is critical to understand the main time trends in child feeding to better explain the observed trends in child's growth outcomes. However, changes over time in Nepali children's diets and in feeding practices have not been examined. Better understanding the determinants of poor complementary feeding at the individual, household, and community levels can also help to understand the epidemiology of poor child nutrition and also assist in tailoring programme interventions (Aguayo & Menon, 2016).

Using nationally representative data from Nepal, this paper focuses on the time trends and predictors of four core WHO‐UNICEF complementary feeding indicators that assess the timely introduction of complementary foods, the frequency of feeding, the diversity of foods, and the overall adequacy of diets among Nepali children aged 6–23 months using survey data collected from 2001 to 2014. The specific aims of the study are (a) to examine time trends in each of the four complementary feeding indicators in all and within subgroups of children; (b) to investigate time trends in food group consumption in children by child age and by survey month; and (c) to identify factors at the individual, household, and community levels that are related to positive complementary feeding practices.

**Key messages**

- Despite rapid improvement in health and development indicators, slow progress has been made in complementary feeding practices in the past 15 years in Nepal with average annual rate of increase ranged from 1% to 2% per year.
- Disparities in the risk of inappropriate complementary feeding practices are significantly evident at both individual (child age, maternal education, antenatal visits, and sociocultural group) and community‐level (ecological zone and community‐level access to health care).
- Contextual socio‐economic progress that has driven improved access to education and health care services did not automatically address poor child feeding practices in Nepal over time.

## 2 METHODS

### 2.1 Study sample

To analyse trends over time, we used data from four nationally representative surveys: the Nepal Demographic and Health Surveys (NDHS) in 2001, 2006, and 2011 and the Nepal Multi‐Indicator Cluster Survey (NMICS) in 2014. Details on the methods for these surveys have been published previously (Central Bureau of Statistics, 2015; Ministry of Health [Nepal] et al., 2002; Ministry of Health and Population et al., 2012; Ministry of Health and Population (MOHP) [Nepal] et al., 2007). The women and children in all four datasets were included based on a two‐stage stratified, nationally representative sample of households. There are 75 administrative districts in Nepal, which are divided into village development committees and then wards. In most cases, the primary sampling unit (PSU) cluster for the surveys was the ward. In rural areas where the ward were small, neighbouring wards were combined into one cluster; whereas in urban areas when the ward size is large, wards were subdivided to form smaller clusters. Due to differences in population census data and PSU definitions between NDHS and NMICS, the number of PSUs selected by systematic sampling techniques varied in 2001 (*n* = 257), 2006 (*n* = 260), 2011 (*n* = 289), and 2014 (*n* = 520). The secondary sampling unit was the household. The average number of households per PSU was between 30 and 40 in NDHS and was 25 in NMICS. The NDHSs and NMICS were conducted between February and August in the survey years. For the purposes of our analysis, our inclusion criteria were (a) youngest children who were singletons; (b) children aged 6–23 months; (c) mothers aged 15–49 years; (d) children alive at the time of survey; and (e) children usually living with their mothers.

### 2.2 Complementary feeding practices

According to the WHO definitions (World Health Organization, 2010), we have defined the four complementary feeding indicators as follows:

*Introduction of solid, semi‐solid, or soft foods (INTRO):* The proportion of infants 6–8 months of age who received solid, semi‐solid, or soft foods in the previous day or night.
*Minimum meal frequency (MMF):* The proportion of breastfed and non‐breastfed children 6–23 months of age who received solid, semi‐solid, or soft foods the minimum recommended number of times or more in the previous day or night. For breastfed children, MMF requires at least two solid/semi‐solid feeds for children aged 6–8 months and at least three feeds for children aged 9–23 months. For non‐breastfed children, MMF is defined as at least four feeds of complementary food or milk between 6 and 23 months of age. MMF is only defined for breastfed children in the NDHS 2006 and 2011 because frequency of milk feeds was not available for non‐breastfed children in these two datasets.
*Minimum dietary diversity (MDD):* The proportion of children 6–23 months of age who received foods from four or more food groups in the previous day or night. Seven food groups were defined as (a) grains, roots, and tubers; (b) legumes and nuts; (c) dairy products; (d) flesh foods; (e) eggs; (f) vitamin‐A‐rich fruits and vegetables; and (g) other fruits and vegetables. In NDHS 2001, the consumption of meat, poultry, fish, shellfish, and eggs were asked in one question, and therefore, MDD could not be created for year 2001. However, to compare food group intake patterns over time, we combined flesh foods and eggs in the other three datasets for analysis.
*Minimum acceptable diet (MAD):* The proportion of children 6–23 months of age who received at least the minimum recommended dietary diversity and the minimum recommended meal frequency in the previous day or night. MAD for breastfed children is defined as meeting MMF and MDD. For non‐breastfed children, MAD is met if the child meets the MMF criterion and receives at least two milk feedings as well as at least four food groups excluding milk products. This indicator is also missing in NDHS 2001 and is confined to breastfed children in NDHS 2006 given missing data on milk feeds.

In summary, data was available for analysis of time trends in INTRO and MMF in years 2001, 2006, 2011, and 2014; and for MDD and MAD, in year 2006, 2011, and 2014. All eligible children were included for sample description. However, some feeding indicators were only available in breastfed children in certain years (MMF in 2001 and 2006; MAD in 2006). To present findings among the same sample, only breastfed children (95–97% of all eligible children) were included for trend analysis.

### 2.3 Risk factors

Risk factors were selected from individual, household, and community levels based on our conceptual framework (Stewart et al., 2013) and data availability. To analyse risk factors associated with the complementary feeding indicators, we did not include NDHS 2001 because of missing key risk factors (e.g., household wealth) and missing feeding indicators of MDD and MAD. Further, we restricted risk factor analysis in breastfed children, whose data was readily available in year 2006, 2011, and 2014 for all four feeding indicators. Table 1 lists the variables and their definitions used in the risk factor analysis. A brief introduction of variables at different levels is provided here.

**Table 1:** List of variables included in the risk factor analysis

	Definition
Child characteristics	
Age (months)	6–11, 12–17, or 18–23
Sex	Female or male
Birth order	Firstborn, second to fourth born, or fifth or higher
Birth interval (month)	No previous birth, <24, or ≥24
Perceived birth weight	Size of child as reported subjectively by the respondent and categorized into smaller than average, average, and larger than average
Vitamin A supplementation	Yes or no, for receiving supplementation in the past 6 months (NDHS 2006 and 2011) or in the past day (NDHS 2014)
Complete age‐appropriate vaccination	Whether or not receiving sufficient doses of BCG, DPT, polio and measles vaccine at proper age per WHO immunization guidelines (World Health Organization). Categorized as none, some, or complete
Child health	
Diarrhoea	Yes or no for any reported diarrhoea in the past 2 weeks
Fever	Yes or no for any reported fever in the past 2 weeks
Cough	Yes or no for any reported cough in the past 2 weeks
Maternal characteristics	
Age (years)	15–24, 25–34, or 35–49
BMI (kg/m^2^)	<18.5, 18.5–24.9, or ≥25
Smoker	Yes or no for current smoking
Utilization of reproductive health care	
Place of delivery	Health facility or other
Type of delivery assistance	Delivered by health professional, traditional birth attendant, or other
Caesarean delivery	Yes or no
Number of antenatal clinic visits	None, 1–3, or ≥4
Timing of postnatal check‐up on woman (days)	0–1, ≥2, or missing/unknown
Timing of postnatal check‐up on child (days)	0–1, ≥2, or missing/unknown
Education	No education, primary, or secondary/higher
Exposure to media	
Reading newspaper	Read at least once a week or less often
Listening to radio	Listened at least once a week or less often
Watching TV	Watched at least once a week or less often
Involved in decision‐making on	
How man's income is used	Yes or no
Large household purchases	Yes or no
Visiting family and friends	Yes or no
Regarding own health care	Yes or no
Attitude toward domestic violence: Beating justified if	
Goes out without telling him	Yes or no
Neglects the children	Yes or no
Argues with him	Yes or no
Refuses to have sex with him	Yes or no
Burns the food	Yes or no
Overall attitude toward domestic violence	High if all five above reported as no, or low if at least one above reported as yes
Women's empowerment score	Total number of “yes” of questions under decision making domain, plus 1 if “high” or plus 0 if “low” in overall attitude toward domestic violence
Sociocultural groups	Brahmin/Chhetri, Janajati, Dalit, or other, according to (Bennett et al., 2008)
Paternal characteristics	
Age (years)	15–24, 25–34, or ≥35
Education	No education, primary, or secondary/higher
Occupation	Categorized into not working, agricultural and non‐agricultural
Household characteristics	
Sex of household head	Female or male
No. of household members	Total number of people living in the same household
No. of children under 5 years	Total number of children under 5 years living in the same household
Types of cooking fuel	Efficient (electricity, LPG, natural gas, and biogas) or non‐efficient fuels (wood, straw/shrubs/grass, animal dung, and other)
Water characteristics	
Source of drinking water	Improved or unimproved according to (World Health Organization & UNICEF, 2006)
Location of drinking water	In own dwelling/yard/plot or elsewhere
Time to get to water source (min)	0, 1–59, or ≥60
Toilet characteristics	
Types of toilet facility	Improved or unimproved according to (World Health Organization & UNICEF, 2006)
Shared toilet with other households	Yes or no
Household wealth	Categorized into quintiles
Community characteristics	
Place of residence	Rural or urban
Development region	Eastern, Central, Western, Mid‐Western, or Far‐Western
Ecological zone	Terai, hill, or mountain
Overall maternal education	Proportion of women within community completed primary or higher education
Overall women's empowerment	Mean women's empowerment score within community
Overall toilet condition	Proportion of households within community using unimproved toilets
Overall shared toilet	Proportion of households within community using shared toilets
Access to health care	
Overall child vaccination status	Proportion of children 0–5 years within community with age‐appropriate vaccination
Overall facility delivery	Proportion of women within community who gave birth to their youngest child 0–5 years at health facilities
Overall health professional delivery	Proportion of women within community who have given birth to their youngest child 0–5 years assisted by health professionals
Overall caesarean delivery	Proportion of women within community who have given birth to their youngest child 0–5 years by caesarean delivery
Overall utility of antenatal clinic visits	Proportion of women within community who had at least four antenatal clinic visits prior to the birth of their youngest child 0–5 years
Overall timing of postnatal check‐up on women	Proportion of women within community who had their postnatal check‐up done within 1 day after delivery of their youngest child 0–5 years
Overall timing of postnatal check‐up on children	Proportion of youngest children 0–5 years within community who had their postnatal check‐up done within 1 day after delivery
Overall child vitamin A supplementation	Proportion of youngest children 0–5 years within community received vitamin A in the past 6 months
Overall child iron supplementation	Proportion of youngest children 0–5 years within community received iron pills, sprinkles or syrup in the past 7 days
Overall maternal iron supplementation	Proportion of women within community given or who bought iron tablets during pregnancy of their youngest children 0–5 years
Rank of access to health care	The summed rank of all community‐level indicators was created as the composite index of overall access to health care. The summed rank was categorized into quintiles.

*Note*. NDHS = Nepal Demographic and Health Surveys; BCG = bacille Calmette‐Guerin; DPT = diphtheria, tetanus, and pertussis; BMI = body mass index.

At the individual level, the following characteristics describing the child's, mother's, and father's attributes were included: child age, sex, birth order, birth interval, perceived birth weight, vitamin A supplementation, vaccination, child diarrhoea, fever, and/or cough in the past 2 weeks; maternal age, body mass index, smoking status, utilization of reproductive health care, maternal education, exposure to media, women's empowerment (Kishor, 2005), sociocultural groups (Bennett, Dahal, & Govindasamy, 2008); and paternal age, education, and occupation.

At the household level, we considered sex of household head, number of household members, number of children under 5 years of age, types of cooking fuel, water, and sanitation characteristics, and household wealth index quintiles that were derived by NDHS or NMICS for relative wealth comparison in the sample using socio‐economic indicators (Rutstein et al., 2004).

At the community level, we include place of residence and two geographic variables: development region (*n* = 5) delineated from east to west and ecological zone (*n* = 3; mountain, hill, and terai). We also calculated the following indicators using information of all survey participants within each PSU: (a) the proportion of women with primary education or higher; (b) mean women's empowerment score; (c) proportion with unimproved toilets; (d) proportion with shared toilets; and (e) general access to health services. This last indicator was a rank score based on 8–10 available variables that describe the utilization of maternal and child nutrition and health care services among all respondents in the cluster. A detailed description of indicator construction is available elsewhere (Na, Aguayo, Arimond, & Stewart, 2017).

### 2.4 Statistical analysis

Using the provided sampling weights and defining strata by geographic region and place of residence, we adjusted for the complex sampling design in NDHS and NMICS to estimate proportions, means and medians that describe the distribution of sample characteristics and complementary feeding indicators at the population level. The Taylor series linearization method was used to estimate confidence intervals around prevalence estimates (Wolter, 2007).

To examine time trends in the entire sample, we have calculated the average annual rate of increase (*AARI*) for proportions that described the sample characteristics, complementary feeding indicators, and food group consumption. *AARI* was calculated to measure the geometric progression ratio, at which proportion changes annually over the period between the first and the latest observed year. Linear or logistic regressions adjusting for complex sampling design were used to test the significance of trends in continuous or binary variables over time. The non‐parametric tests were performed to test the significance of trends in ordinal variables over year.

To study the time trend in complementary feeding indicators in subgroups of children, we calculated the weighted proportion and 95% confidence intervals of children meeting indicator criteria by selected sample characteristics. To test if rates of change in complementary feeding indicators differed in subgroups of the sample, an interaction term was created between the group and year and was added to the logistic regression model for each feeding indicator. The slopes representing the linearized rates were estimated, plotted, and compared against each other by contrasting the marginal effects. Delta methods were used to determine statistical significance (Cameron & Trivedi, 2005).

To identify factors associated with complementary feeding, we applied multilevel models to account for the multistage structure in NDHS and NMICS. We first applied intercept only models to understand the source of variance at individual/household (Level 1) and community (Level 2) levels. Before proceeding, we applied log likelihood ratio tests to compare if the single‐level random intercept models at Level 1 were significantly improved from intercept only models. Next, we examined bivariate associations between risk factors and the four complementary feeding indicators in 2006, 2011, and 2014 by applying two‐level random intercept only models at both Level 1 and Level 2 for each pair of risk factor and outcome of interest. The risk factors that showed significant bivariate associations were included in the multivariable two‐level logistic models to identify independent risk factors in each year. We removed variables with variance inflation factors greater than five to avoid potential collinearity. In the pooled analysis using data from 2006, 2011, and 2014, we included variables from the final year‐specific models and reran the multivariable analyses, including a dummy year variable. To understand potential bias introduced in multilevel models, we examined the intra‐class correlation in each community‐level attribute in each year and in pooled data and compared with the recommended cut‐off of ≥0.2 (Kravdal, 2006). Finally, we conducted a series of sensitivity analyses using alternative analytic approaches to test the robustness of the results, including (a) constructing multivariable multilevel models using backward stepwise selection at *p* = 0.1 level; (b) fixing several confounder variables (child sex, maternal, and paternal age) in the multivariable models; (c) repeating bivariate and multivariable analysis using logistic regression models ignoring the clustering effect; and (c) repeating analysis in all children (both breastfed and non‐breastfed) given data availability. We used stata/*SE* 14.1 (StataCorp, College Station, TX) to analyse data, and values of *p* < 0.05 were considered significant for all tests.

## 3 RESULTS

### 3.1 Time trends in sample characteristics

Table 2 presents the characteristics of the study sample at individual, household, and community levels in 2001, 2006, 2011, and 2014. Girls represented about half of the sample, although the proportion was lower in 2014. Almost all children were still breastfed in all survey rounds (95–98%, *p*‐trend = 0.08). From 2001 to 2014, there was a declining prevalence of diarrhoea (32% to 18%, *AARI* = −4.1%), fever (44% to 25%, *AARI* = −4.1%), and cough (53% to 28%, *AARI* = −4.7%, all *p*‐trend < 0.001). The prevalence of maternal undernutrition (body mass index < 18.5 kg/m^2^) also showed a decreasing trend from 27% in 2001 to 23% in 2011 (*AARI* = −1.7%, *p*‐trend < 0.001). Utilization of health care services increased by three‐ to fivefold from 2001 to 2014 in terms of the proportion of women delivering in a health facility (11% to 56%, *AARI* = 13.6%), delivering with skilled assistance (13% to 49%, *AARI* = 10.7%), and benefitting from at least four antenatal clinic visits (15% to 70%, *AARI* = 12.8%, all *p*‐trend < 0.001). More than 60% and 80% of women and their partners, respectively, completed at least primary education in 2014, with significant improvements since 2001 (28% to 62%, *AARI* = 6.3% for women; 65% to 81%, *AARI* = 1.7% for men; both *p*‐trend < 0.001). At the household level, the proportion of female‐headed households increased (11% to 25%, *AARI* = 6.3%, p‐trend < 0.001); the proportion with access to unimproved water decreased (25% to 7.2%, *AARI* = −9.2%, *p*‐trend < 0.001); and the proportion with unimproved sanitation decreased between 2001 and 2014 (77% to 32%, *AARI* = −6.6, *p*‐trend < 0.001). At the community level, the majority of households remained in rural areas, although there was a significant decreasing trend in rural residence (85% to 77%, *AARI* = −0.7%, *p*‐trend < 0.05).

**Table 2:** Demographic and socio‐economic characteristics from 2001 to 2014 in Nepal

	2001		2006		2011		2014		*AARI* a (%)	*p*‐trend^b^
	*N*	% or mean (*SE*)	*N*	% or mean (*SE*)	*N*	% or mean (*SE*)	*N*	% or mean (*SE*)		
Child characteristics										
Female	1885	52.3	1418	48.2	1431	50.6	1471	46.8	−0.9	0.03
Currently breastfed	1885	97.0	1418	97.5	1431	95.3	1471	96.3	−0.1	0.08
Age (months)	1885		1418		1431		1471			0.70
6–11		33.3		34.5		33.8		34.3	0.2	
12–17		33.5		32.8		35.9		31.4	−0.5	
18–23		33.1		32.7		30.3		34.3	0.3	
Child morbidity in the past 2 weeks:										
Diarrhoea	1884	31.7	1418	20.7	1431	23.6	1470	18.3	−4.1	<0.001
Fever	1883	43.8	1418	24.1	1431	26.0	1471	25.2	−4.1	<0.001
Cough	1884	52.7	1418	25.3	1431	30.3	1471	28.2	−4.7	<0.001
Maternal characteristics										
Age (years)	1885	26.4 (0.19)	1418	25.5 (0.21)	1431	25.6 (0.17)	1471	25.7 (0.18)	—	0.01
BMI (kg/m^2^) <18.5	1882	27.3	1410	26.6	705	23.1	—	—	−1.7	<0.001
Delivered at health facility	1885	10.5	1418	18.8	1431	43.3	1469	55.5	13.6	<0.001
Delivered by health professional	1885	12.9	1418	20.5	1431	43.1	1468	48.5	10.7	<0.001
Antenatal clinic visit ≥4	1885	14.6	1418	30.7	1431	55.1	1284	70.3	12.8	<0.001
Primary or higher education completed	1885	28.1	1418	43.9	1431	58.2	1468	61.9	6.3	<0.001
Paternal characteristics										
Age (years)	1872	30.8 (0.24)	1404	29.5 (0.27)	1420	29.9 (0.25)	1437	29.7 (0.22)	—	0.002
Primary or higher education completed	1839	65.0	1411	78.4	1428	78.8	1021	80.5	1.7	<0.001
Household characteristics										
Female household head	1885	11.4	1418	18.9	1431	24.0	1471	25.1	6.3	<0.001
No. of HH members	1885	7.0 (0.10)	1418	7.0 (0.28)	1431	6.3 (0.23)	1437	6.4 (0.16)	—	<0.001
No. of children under 5 years	1885	1.9 (0.03)	1418	1.9 (0.06)	1431	1.6 (0.05)	1471	1.6 (0.04)	—	<0.001
Unimproved source of drinking water	1884	25.3	1418	18.6	1431	10.4	1471	7.2	−9.2	<0.001
Unimproved toilet facility	1885	76.6	1416	69.1	1431	56.1	1469	31.5	−6.6	<0.001
Shared toilet with other households	1841	86.2	1389	72.3	1369	64.5	1470	58.0	−3.0	<0.001
Community characteristics										
Rural residence	246	85.0	252	69.0	280	68.6	489	77.1	−0.7	0.04
Development region	246		252		280		489			<0.001
Eastern		26.4		23.4		23.6		20.4	−2.0	
Central		30.1		26.6		25.7		25.4	−1.3	
Western		18.3		19.0		22.5		16.4	−0.9	
Mid‐Western		11.4		15.9		15.7		19.0	4.0	
Far‐Western		13.8		15.1		12.5		18.8	2.4	

*AARI* = annual average rate of increase for proportions; *SE* = standard error.

a *AARI* is calculated as [(% in the latest year/% in the earliest year)^(1/difference between latest year and earliest year)] – 1.

b *p*‐trend values are from linear or logistic regression between continuous or binary sample characteristics and year adjusting for complex sampling design. *p*‐trend values for ordinal variables are from non‐parametric tests.

### 3.2 Time trends in complementary feeding practices in all children

**Figure 1:**
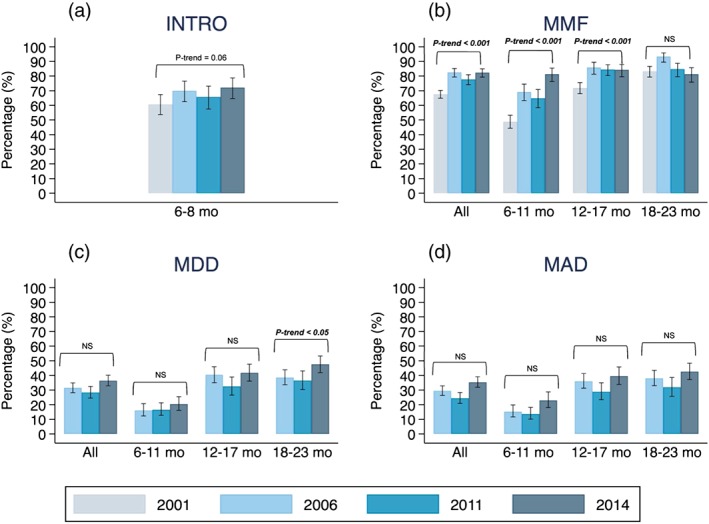
Trends of complementary feeding in children aged 6–23 months in Nepal from 2001 to 2014. (a) Introduction of solid, semi‐solid or soft foods (INTRO); (b) minimum meal frequency (MMF); (c) minimum dietary diversity (MDD); and (d) minimum acceptable diet (MAD). Only breastfed children are included. Error bars represent the 95% confidence intervals of the weighted proportion. MDD and MAD is missing for year 2001 because the consumption of meat, poultry, fish, shellfish, and eggs were asked in one question. NS = not significant or *p*‐trend value > 0.05

Figure 1 displays the prevalence of complementary feeding indicators by age. From 2001 to 2014, the weighted proportion of children who had received complementary foods at 6–8 months of age increased (61% to 72%, *AARI* = 1.3%, *p*‐trend = 0.06). The proportion of children who met the MMF criteria also increased (68% to 82%, *AARI* = 1.5%, *p*‐trend <0.001). The weighted proportion (95%CI) of children meeting the MDD criteria was 31.5% [28.2, 34.9], 28.3% [24.5, 32.4], 36.4% [32.9, 40.2] in 2006, 2011, and 2014 (*AARI* = 1.8%, *p*‐trend = 0.11). The proportion of children meeting the MAD criteria was 29.5% [26.3, 32.8], 24.4% [20.9, 28.4], and 35.4% [31.9, 39.1] in the same years (*AARI* = 2.3%, *p*‐trend = 0.15).

### 3.3 Time trends in complementary feeding practices in population subgroups

We examined time trends in complementary feeding indicators by individual, household, and community characteristics. Only significant results are presented by child sex, child age group, and maternal age group in Figures S1–S3. The proportion of girls meeting the INTRO criteria stagnated at ~65% between 2001 and 2014 whereas in boys, the proportion steadily increased from 55% to 81% in the same period (Figure S1, difference in slope *p*‐value < .01, *AARI* = 3.0%). Considering child age (Figure S2), the proportion of children meeting the MMF criteria increased most rapidly among children aged 6–11 months (from 49% to 82%, *AARI* = 4.0%) followed by children aged 12–17 months (from 72% to 85%, *AARI* = 1.3%). Both rates exceeded that of children aged 18–23 months, who stayed at a high proportion around 80–90% (all pairwise slope comparison *p*‐values < .01). Children whose mothers were in the youngest age group (15–24 years old), showed significantly worse MDD and MAD trends over time (Figure S3); both proportions dropped from ~30% to ~20% (*AARI* = −4.8% for MDD; −5.6% for MAD, pairwise slope comparison against older groups: all *p*‐value < 0.05). This stands in sharp contrast to the generally improving trends among children of older mothers.

### 3.4 Time trends in dietary diversity and food group consumption

**Figure 2:**
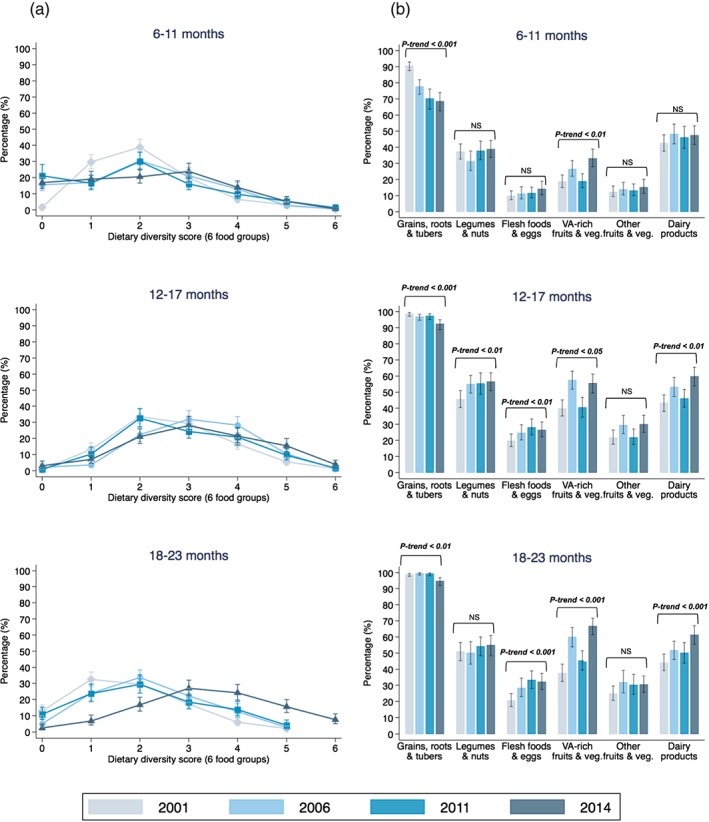
Trends in child dietary diversity score (a) and food group consumption (b) from 2001 to 2014 by child age. VA = vitamin A. *p*‐trend in the weighted mean dietary diversity score for children aged 6–11, 12–17, and 18–23 months is 0.58, 0.10, and 0.06, respectively. NS = not significant or *p*‐trend value > 0.05

Figure 2 presents the distribution of child dietary diversity scores based on six food groups (Panel A) and the proportion of individual food groups consumed (Panel B) by child age group. In children aged 6–11 months, the weighted mean (*SE*) food group score based on six food groups were 2.1 (0.05), 2.1 (0.08). 2.0 (0.11), and 2.2 (0.09) in 2001, 2006, 2011, and 2014, respectively (*p*‐trend = 0.58). Despite the similar overall means, the proportion of children who were not fed any complementary foods were significantly higher in 2006, 2011, and 2014 (15.5–21.1%) than in 2001 (1.5%) (*p* < .05). Children aged 12–17 months consumed on average (mean [SE]) three out of six food groups between 2001 and 2014 (2001: 2.7 [0.06]; 2006: 3.1 [0.06]; 2011: 2.9 [0.08]; 2014: 3.2 [0.08], *p*‐trend = 0.10). The average score increased marginally in children aged 18–23 months during the same period (2001: 2.8 [0.06]; 2006: 3.2 [0.07]; 2011: 3.1 [0.09]; 2014: 3.4 [0.08], *p*‐trend = 0.06).

Consumption of grains, roots, and tubers was nearly universal and was the highest among the six food groups regardless of child age and year of survey; however, there was a decreasing trend in feeding children grains, roots, and tubers, most evident in the youngest age group from 2001 (91%) to 2014 (69%) (*p*‐trend < 0.001). In general, the consumption of the other five food groups increased over time and with child age. The proportion of children who consumed vitamin‐A‐rich fruits and vegetables increased the most, from 19% to 33% among children aged 6–11 months (*p*‐trend < 0.01), from 40% to 56% in children aged 12–17 months (*p*‐trend < 0.05), and from 37% to 67% in children aged 18–23 months (*p*‐trend < 0.001). The consumption of vitamin‐A‐rich fruits and vegetables was much higher in May 2006 (>60%) than in the same month of other years (25–50%), which did not follow the seasonal pattern and may have driven some of the increase observed in 2006 (Figure S4). The increase in the proportion of children who consumed dairy foods was smaller: from 43% to 47% in children aged 6–11 months (*p*‐trend = 0.33), from 43% to 60% in children aged 12 to 17 months (*p*‐trend < 0.01), and from 45% to 61% in children aged 18–23 months (*p*‐trend < 0.001). The proportion of children who were fed flesh foods and eggs increased moderately over time, from 17% in 2001 to 24% in 2014 across all age groups (*p*‐trend < 0.001). Yet in 2014 the proportion children consuming any flesh foods or eggs was still quite low at 14%, 27%, and 32% in the three age groups, respectively. There were no significant changes among all children in the consumption of legumes and nuts (*p*‐trend = 0.06) and other fruit and vegetables from 2001 to 2014 (*p*‐trend = 0.11).

### 3.5 Independent factors associated with positive complementary feeding indicators

Variables included in the final year‐specific multivariable models, and the estimated odds ratios in relation to complementary feeding indicators were comparable in 2006, 2011, and 2014 (data not shown). Table 3 presents the independent factors associated with appropriate complementary feeding practices in breastfed children by pooling data from 2006 to 2014. Using pooled data, only factors that remained statistically significant to predict at least one complementary feeding indicators in the final models are presented in Table 3. Comparing to 2006, the odds of meeting MMF, MDD, and MAD decreased by 39–44% in 2011 after accounting for sample characteristics.

**Table 3:** Determinants of meeting the complementary feeding practice criteria in breastfed children in Nepal from 2006 to 2014 using multivariable multilevel logistic regression analysis^a^

	INTRO			MMF			MDD			MAD		
	Estimate		*p*‐value	Estimate		*p*‐value	Estimate		*p*‐value	Estimate		*p*‐value
	OR	(95%CI)		OR	(95%CI)		OR	(95%CI)		OR	(95%CI)	
*N*		697			3,715			3,795			3,698	
Year												
2006	1.00	(Referent)		1.00	(Referent)		1.00	(Referent)		1.00	(Referent)	
2011	0.79	[0.49, 1.28]	.34	0.56	[0.42, 0.74]	<.001	0.67	[0.53, 0.86]	.001	0.61	[0.50, 0.75]	<.001
2014	0.63	[0.33, 1.20]	.16	0.87	[0.62, 1.21]	.40	0.97	[0.74, 1.27]	.84	1.01	[0.78, 1.30]	.95
Child characteristics												
Age (months)												
6–11				1.00	(referent)		1.00	(Referent)		1.00	(Referent)	
12–17				2.42	[1.94, 3.01]	<.001	3.63	[2.96, 4.46]	<.001	3.18	[2.58, 3.92]	<.001
18–23				3.20	[2.52, 4.05]	<.001	4.44	[3.60, 5.48]	<.001	3.82	[3.09, 4.72]	<.001
Birth order												
Firstborn	1.64	[1.03, 2.62]	.04	0.96	[0.75, 1.23]	.75	1.20	[0.98, 1.48]	.08	1.11	[0.91, 1.37]	.31
Second to fourth	1.00	(Referent)		1.00	(Referent)		1.00	(Referent)		1.00	(Referent)	
Fifth and more	1.30	[0.71, 2.39]	.40	0.98	[0.71, 1.35]	.90	1.25	[0.92, 1.69]	0.16	1.27	[0.93, 1.73]	.13
Perceived birth weight												
Smaller than average				0.92	[0.72, 1.16]	.47	0.79	[0.63, 0.99]	.04	0.82	[0.66, 1.03]	.09
Average				1.00	(Referent)		1.00	(Referent)		1.00	(Referent)	
Larger than average				1.08	[0.85, 1.37]	.55	0.98	[0.81, 1.20]	.85	0.97	[0.79, 1.18]	.76
Maternal characteristics												
Age (years)												
15–24				0.82	[0.59, 1.13]	.22	0.75	[0.56, 1.00]	.05	0.79	[0.59, 1.06]	.11
25–34				1.00	(Referent)		1.00	(Referent)		1.00	(Referent)	
35–49				0.79	(0.58, 1.09)	.16	0.91	[0.68, 1.21]	.51	0.83	[0.63, 1.11]	.21
Antenatal clinic visits												
None	0.73	[0.36, 1.46]	.37	0.68	[0.49, 0.94]	.02	0.59	[0.43, 0.81]	.001	0.57	[0.41, 0.78]	.001
1–3	1.17	[0.74, 1.86]	.51	0.78	[0.62, 0.98]	.03	0.76	[0.63, 0.93]	.01	0.74	[0.61, 0.90]	.003
≥4	1.00	(Referent)		1.00	(Referent)		1.00	(Referent)		1.00	(Referent)	
Postnatal check‐up on woman												
0–1 day				1.00	(Referent)		1.00	(Referent)		1.00	(Referent)	
≥ 2 days				0.76	[0.46, 1.28]	.31	0.84	[0.54, 1.29]	.42	0.76	[0.49, 1.18]	.22
Missing or unknown				0.87	[0.68, 1.13]	.30	0.76	[0.61, 0.95]	.01	0.78	[0.63, 0.98]	.03
Education												
No education	1.00	(Referent)		1.00	(Referent)		1.00	(Referent)		1.00	(Referent)	
Primary	1.15	[0.68, 1.94]	.61	1.30	[1.00, 1.70]	.05	1.13	(0.89, 1.44)	.31	1.15	[0.90, 1.47]	.25
Secondary or higher	2.46	[1.43, 4.25]	.001	1.64	[1.25, 2.15]	<0.001	2.43	(1.93, 3.04)	<.001	2.32	[1.84, 2.92]	<.001
Sociocultural group												
Brahmin/Chhetri	1.00	(Referent)		1.00	(Referent)		1.00	(Referent)		1.00	(Referent)	
Janajati	0.94	[0.55, 1.61]	.82	0.91	[0.70, 1.19]	.50	0.60	[0.48, 0.75]	<.001	0.65	[0.52, 0.81]	<.001
Dalit	0.55	[0.31, 0.97]	.04	0.79	[0.60, 1.06]	.12	0.57	[0.44, 0.74]	<.001	0.64	[0.50, 0.83]	.001
Other	0.40	[0.21, 0.77]	.01	0.67	[0.48, 0.94]	.02	0.64	[0.48, 0.85]	.002	0.63	[0.47, 0.84]	.002
Community characteristics												
Geographical region												
Eastern	1.00	(Referent)		1.00	(Referent)		1.00	(Referent)		1.00	(Referent)	
Central	0.60	[0.34, 1.07]	.08	0.77	[0.54, 1.10]	.15	0.83	[0.62, 1.12]	.22	0.81	[0.61, 1.08]	.16
Western	0.75	[0.41, 1.40]	.37	0.95	[0.65, 1.38]	.78	1.11	[0.83, 1.49]	.48	1.06	[0.80, 1.41]	.69
Mid‐Western	1.09	[0.59, 2.02]	.77	0.68	[0.47, 0.99]	.05	0.95	[0.69, 1.29]	.72	0.86	[0.64, 1.17]	.34
Far‐Western	0.46	[0.24, 0.88]	.02	0.56	[0.38, 0.84]	.01	0.88	[0.63, 1.22]	.43	0.84	[0.61, 1.16]	.29
Ecological zone												
Terai	1.00	(Referent)		1.00	(Referent)		1.00	(Referent)		1.00	(Referent)	
Hill	2.69	[1.63, 4.45]	<.001	1.82	[1.38, 2.40]	<.001	1.57	[1.25, 1.96]	<.001	1.61	[1.29, 2.01]	<.001
Mountain	4.17	[2.18, 7.99]	<.001	2.01	[1.42, 2.86]	<.001	1.56	[1.17, 2.08]	.003	1.64	[1.23, 2.18]	.001
Rank of access to health care												
Highest (best access)	1.00	(Referent)		1.00	(Referent)		1.00	(Referent)		1.00	(Referent)	
Higher	0.52	[0.25, 1.08]	.08	0.87	[0.59, 1.30]	.51	0.88	[0.65, 1.18]	.38	1.06	[0.79, 1.42]	.72
Medium	0.80	[0.38, 1.70]	.57	0.94	[0.64, 1.40]	.77	0.89	[0.66, 1.20]	.45	1.00	[0.74, 1.34]	.99
Lower	0.72	[0.34, 1.52]	.39	0.75	[0.51, 1.11]	.15	0.58	[0.43, 0.78]	<.001	0.69	[0.51, 0.93]	.02
Lowest (worse access)	0.44	[0.20, 0.99]	.05	0.73	[0.48, 1.11]	.14	0.40	[0.28, 0.56]	<.001	0.49	[0.35, 0.69]	<.001

INTRO = introduction of solid, semi‐solid, and soft foods; MMF = minimum meal frequency; MDD = minimum dietary diversity; MAD = minimum acceptable diet; OR = odds ratio; CI = confidence interval.

a . Year 2001 was excluded because of missing MDD and MAD. *p*‐values are from the Wald tests.

At the individual level, child older age was consistently and dose‐responsively associated with increased odds of meeting the MMF, MDD, and MAD criteria. Compared to women who had four or more antenatal clinic visits, mothers who had none or 1–3 visits were 22–43% less likely to have children who met the MMF, MDD, and MAD feeding criteria; however, the number of antenatal clinic visits was not associated with INTRO. Compared to mothers with no education, mothers who had secondary or higher education were 1.6–2.5 times more likely to have children who met the INTRO, MMF, MDD, and MAD feeding criteria. Compared to the dominant Brahmin/Chhetri sociocultural group, the Janajati group was less likely to meet the MDD and MAD criteria and the Dalit group was less likely to meet the INTRO, MDD, and MAD criteria. Finally, the “other” sociocultural group was less likely to meet all four complementary feeding criteria. In addition, children who were perceived small at birth (compared to children perceived to have an average or larger birth size), and children of mothers 15–24 years old (compared to children of mothers 25–34 years old) were significantly less likely to meet the MDD feeding criteria (all *p* < .05). No paternal factor was significant in the final models.

None of the household‐level factors remained significant after including community‐level factors in the model. At the community level, using the Terai region as the reference, children living in the hill and mountain areas were ~three to four times more likely to be fed complementary foods at 6–8 months of age (i.e., INTRO) and were ~two times more likely to meet the MMF, MDD, and MAD feeding criteria. The odds of meeting the INTRO and MMF feeding criteria were 54% (12%, 76%) and 44% (16%, 62%) lower, respectively, among children living in in the Far‐Western region as compared to the Eastern region. Compared to communities with better access to health care services, those with the lowest access to health care had significantly lower odds of meeting INTRO, MDD, and MAD criteria.

## 4 DISCUSSION

Using nationally representative data from four survey years, we examined time trends and independent predictors of appropriate complementary feeding practices in Nepal from 2001 to 2014. Over the past decade, complementary feeding practices in Nepal have improved slowly, with *AARI*s ranging from 1% to 2% per year for the four complementary feeding indicators. In contrast to the slow change in feeding indicators, there have been remarkable improvements in maternal education (*AARI* = 6.3%) and access to health care (*AARI* > 10% for the use of health care services) over the same period, which were also documented previously using national data (Ministry of Health and Population Nepal et al., 2014; World Bank, 2016). Taken together, this suggests that complementary feeding practices have not kept pace with improvements in education and access to health care in Nepal over time. Stagnation in complementary feeding indicators were also observed in Bangladesh (2004–2011; Hanif, 2013), which may indicate broader challenges in improving complementary feeding practices across the South Asia region.

Disparities in time trends for complementary feeding practices are evident in certain subgroups of children. While it is common to introduce solid foods at 6 months with a “rice ceremony” (Locks et al., 2015), there were notably different time trends in the introduction of complementary foods among boys and girls. There were much greater rates of improvement over time among boys than among girls. The difference is not likely due to a gender preference because the probability of meeting MMF, MDD, and MAD, for boys and girls has been similar over the past 15 years. Qualitative studies in the 1980s reported that mothers provided complementary foods sooner to boys than to girls in northwestern Nepal, which coincided with mothers' frequent concerns over insufficient breastmilk for boys (Miller, 1997). Given that the time trend has shifted, more emphasis should be placed on ensuring that girls are also receiving a timely introduction of complementary foods.

When examining time trends by child age, we see that children aged 6–11 months and 12–17 months have caught up with children aged 18–23 months in meeting the MMF criterion. The improvement is encouraging and may be attributable to the effectiveness of recent nutrition and health education programmes with emphasis on young children. However, no improvements were found in MDD and MAD in children under 2 years of age. In the general population over roughly the same period, there has been a 31% reduction in the proportion of people consuming less than the minimum dietary energy requirement (United Nations Development Programme in Nepal, 2017). Household dietary diversity has also improved, with a tripling of the consumption of vegetables, more than doubling of consumption of meat and fish, and over 50% increase in fruits consumption (National Planning Commission and Central Bureau of Statistics, 2013). This apparent disparity between the general population improvements in food security and dietary diversification at the household level, yet only modest improvements in infant feeding practices may be due to several reasons. First, there is a widespread cultural belief that cereal foods are enough for children, with less emphasis on the importance of diet diversity for healthy growth and development (Gautam, Adhikari, Khatri, & Devkota, 2016). Second, cultural taboos relating to eggs and flesh foods are common, and children are usually fed such foods only after they have teeth, at about 1 year of age (Locks et al., 2015).

For children of mothers less than 24 years of age, we observed a negative trend in meeting MDD and MAD criteria between 2006 and 2014. Younger maternal age also significantly predicted lower odds of meeting the MDD criterion, after controlling for other individual, household, and community factors. Other observational studies in Nepal have identified young maternal age as a risk factor for inappropriate feeding practices (Joshi, Agho, Dibley, Senarath, & Tiwari, 2012; Khanal, Sauer, & Zhao, 2013). Although there has been significant effort to reduce child marriage, leading to a substantial reduction since 1991 (Raj, McDougal, & Rusch, 2012), child marriage is still prevalent in Nepal, occurring in more than half of girls under 18 years old in 2011 (Raj et al., 2012). While a continued effort to reduce child marriage and early childbearing is paramount, directed efforts to empower young mothers with education and messages about appropriate child feeding practices are also necessary. Our study clearly indicates that programmes need to strengthen efforts to prevent early marriage and early childbearing while improving younger mothers' knowledge and practice with respect to complementary feeding.

Other than maternal and child age, our study points to a few additional consistent and independent factors that predict three or more complementary feeding indicators, including the number of antenatal care visits, maternal education, sociocultural group, ecological zone, and access to health care. Both antenatal care visits and maternal education have been previously identified as strong predictors of complementary feeding practices in Nepal (Dibley et al., 2010; Joshi et al., 2012; Pandey, Tiwari, Senarath, Agho, & Dibley, 2010). We also found that community‐level access to health care was an important predictor of adequate complementary feeding. We have previously shown predictive power of a similarly composed indicator in Pakistan (Na et al., 2017). When mothers have better access to health services for themselves and their children in their communities, this may also reflect greater access to health and nutrition information.

Greater maternal education and better feeding knowledge alone are not likely to fully address poor complementary feeding practice, however (Chapagain, 2013). The Dalit and “other” category of sociocultural groups were less likely to meet the minimum complementary feeding criteria. The “other” category is a diverse group of smaller ethnic and religious castes. The fact that this group had consistently poorer practices might be a reflection on their minority status within communities and that they may have lower access to services and culturally tailored information. As complementary feeding practices are embedded in economic and sociocultural contexts, the challenge is to improve the quality of complementary foods and feeding practices by empowering and enabling caregivers to access and utilize healthier foods.

In this study, we identified a few community‐level indicators that may be useful in identifying at‐risk children. The Terai region shows poorer indicators of appropriate complementary feeding in comparison with other ecological regions (Dibley et al., 2010; Khanal et al., 2013; Pandey et al., 2010), despite the fact that this is a more agriculturally productive zone than the remote mountain and hill regions. We noted that including the indicator of community‐level health care services essentially “mutes” all household factors in predicting feeding practices, including household wealth, which has frequently been cited as a significant predictor of feeding practices in South Asia (Senarath et al., 2012). Although community‐level socio‐economic status was not assessed or examined, this finding has two implications: First, poorer households may cluster within villages, and therefore, targeting and intervening within communities may be more efficient and effective; second, community factors may outweigh household‐level socio‐economic factors in predicting complementary feeding practices.

There are certain limitations to the study. First, secular trends were built on survey data from two different sources. Although all were nationally representative, surveys were designed and conducted differently with respect to sample selection (e.g., sampling units were defined differently), questionnaire design (e.g., food items included were different), and administration (e.g., survey months, training, and data quality control done by NDHS and NMICS were different). Second, analysis of factors was based on pooled cross‐sectional data, and our results only imply associations rather than causal relationships. Third, the NDHS and NMICS surveys were not designed for analysing determinants of complementary feeding practices; therefore, the factors included in the analysis did not cover the all the key immediate, underlying, and basic risk factors. This lack of information may be even more problematic at the community level, for which we did not have detailed information about the dynamics of political, economic, agricultural, and sociocultural factors over time that deeply influence and shape feeding behaviours. Despite these limitations, we have applied multiple nationally representative data over the past 15 years. Rigorous statistical methods were applied to appropriately address the multistage structure, collinearity, and bias in estimation in the multilevel data. We conducted sensitivity analyses and found little difference in the results, which also lends confidence of the robustness of these research findings.

In conclusion, slow progress has been made in complementary feeding practices in the past 15 years, despite progress in numerous other health and development indicators in Nepal. Poor child dietary diversity is affecting 7 in 10 children under two currently and will continue to affect more than 50% of children in 2025 under the current trajectory of progress. Improvement in the access to education, health, and better sanitation did not necessarily translate into improvement in complementary feeding, particularly dietary diversity, which, in turn, did not and will not completely solve the child malnutrition problem. To promote child growth, our study calls for specific programming effort to address poor child dietary diversity in vulnerable and diverse populations in Nepal, with contextual understanding of the drivers of poor child diet and deliverable approaches to improve feeding behaviours.

## CONFLICTS OF INTEREST

The authors declare that they have no conflicts of interest.

## CONTRIBUTIONS

MN, VMA, MA, and CPS conceptualized the research question. MN requested data, conducted literature review, data analysis, and prepared the first draft of the manuscript. VMA, MA, PD, BL, RP, SC, and CPS provided technical support on study methods and insights on results interpretation. All authors read and approved the final manuscript.

## Supporting information


**Figure S1:** Estimated proportion of appropriate complementary feeding practices over time by child sex. Pairwise slope comparison by delta methods: **, *p* < 0.01.
**Figure S2:** Estimated proportion of appropriate complementary feeding practices over time by child age. Pairwise slope comparison by delta methods: **, p < 0.01.
**Figure S3:** Estimated proportion of appropriate complementary feeding practices over time by maternal age. Pairwise slope comparison by delta methods: *, *p* < 0.05; **, *p* < 0.01.
**Figure S4:** Proportion of vitamin A‐rich fruits and vegetables intake by year and by months of survey
**Figure S5:** Proportion of children consumed flesh foods and eggs between 2006 to 2014 by child age
