# Supplementary material for: Trends and predictors of appropriate complementary feeding practices in Nepal: An analysis of national household survey data collected between 2001 and 2014
Source: Matern Child Nutr. 2017 Nov 17;14(Suppl 4):e12564. doi: 10.1111/mcn.12564 (PMC6586161; doi:10.1111/mcn.12564)

**Supplemental Figure 1:** Estimated proportion of appropriate complementary feeding practices over time by child sex. Pairwise slope comparison by delta methods: **, p<0.01.
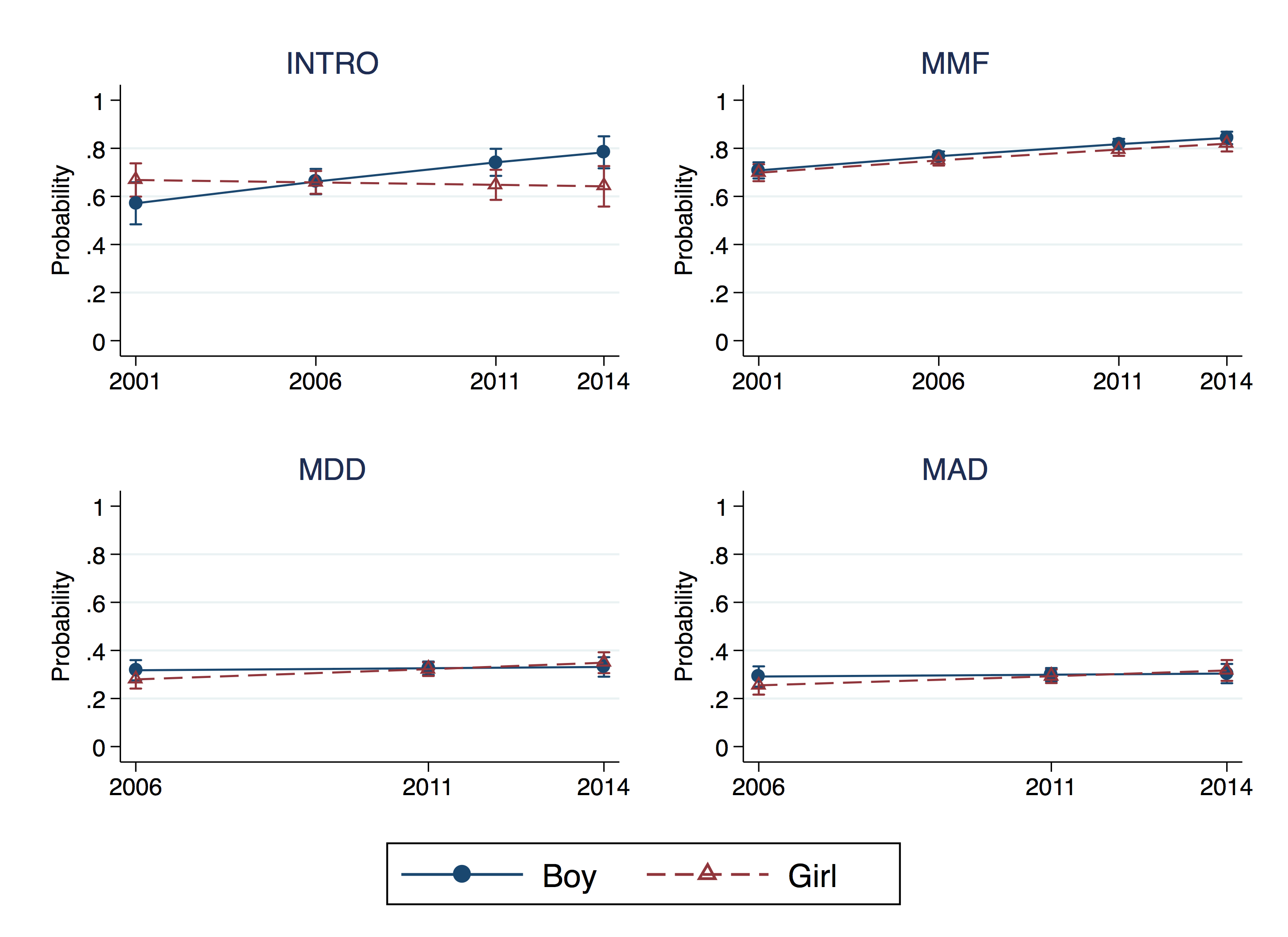


********

**Supplemental Figure 2:** Estimated proportion of appropriate complementary feeding practices over time by child age. Pairwise slope comparison by delta methods: **, p<0.01.


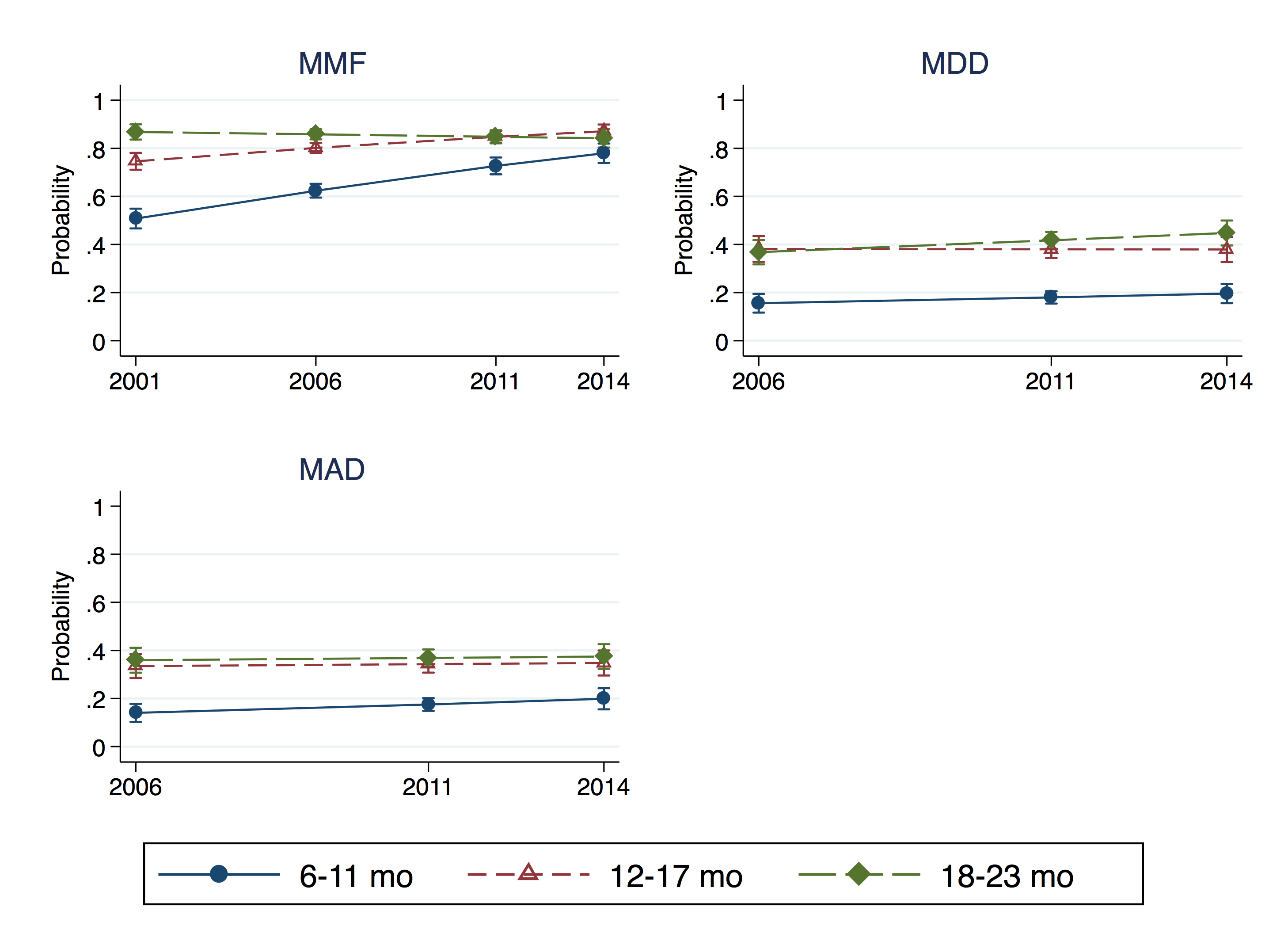


********

**Supplemental Figure 3:** Estimated proportion of appropriate complementary feeding practices over time by maternal age. Pairwise slope comparison by delta methods: *, p<0.05; **, p<0.01.


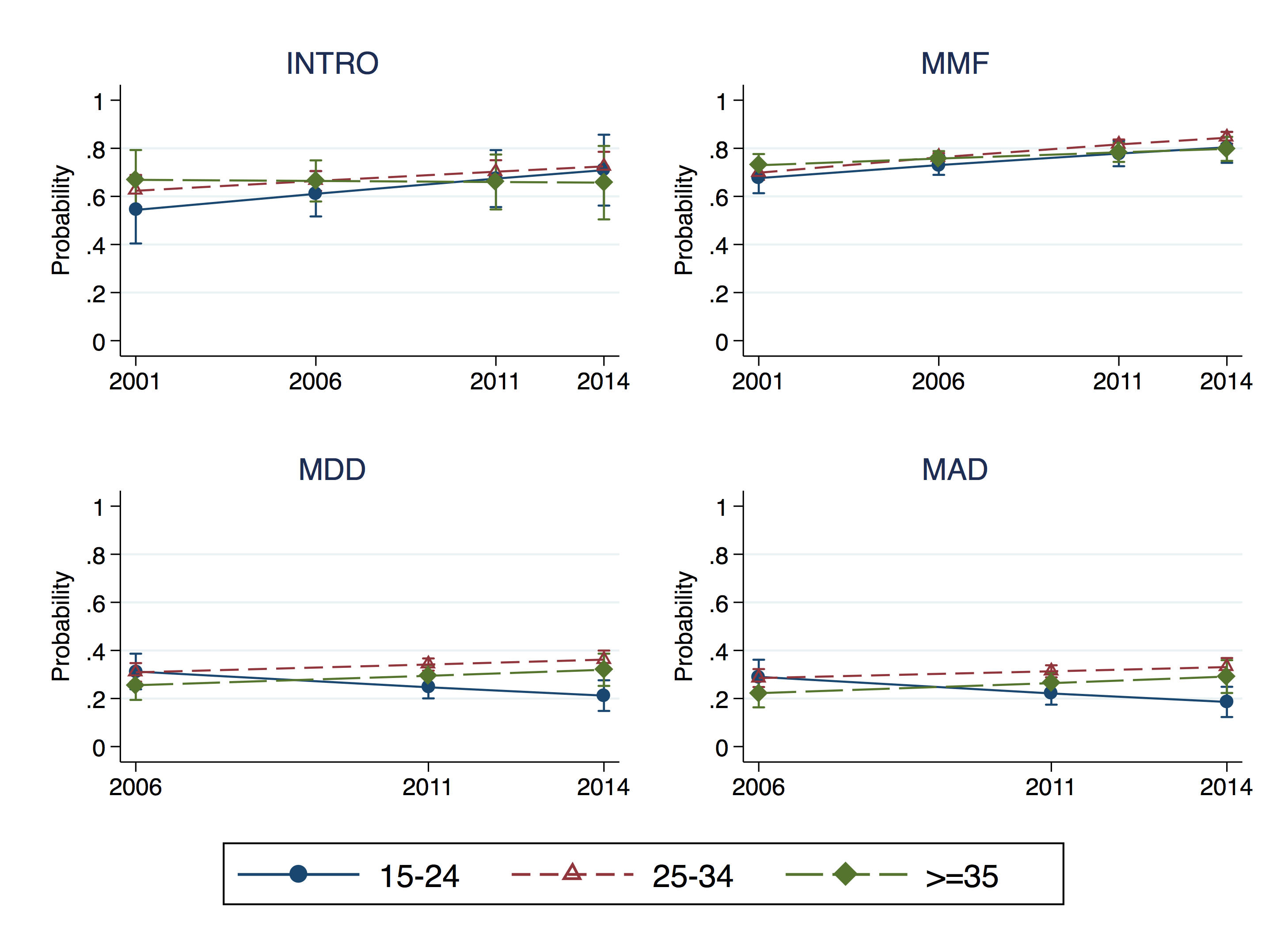


*******

********

********

**Supplemental Figure 4:** Proportion of vitamin A-rich fruits and vegetables intake by year and by months of survey


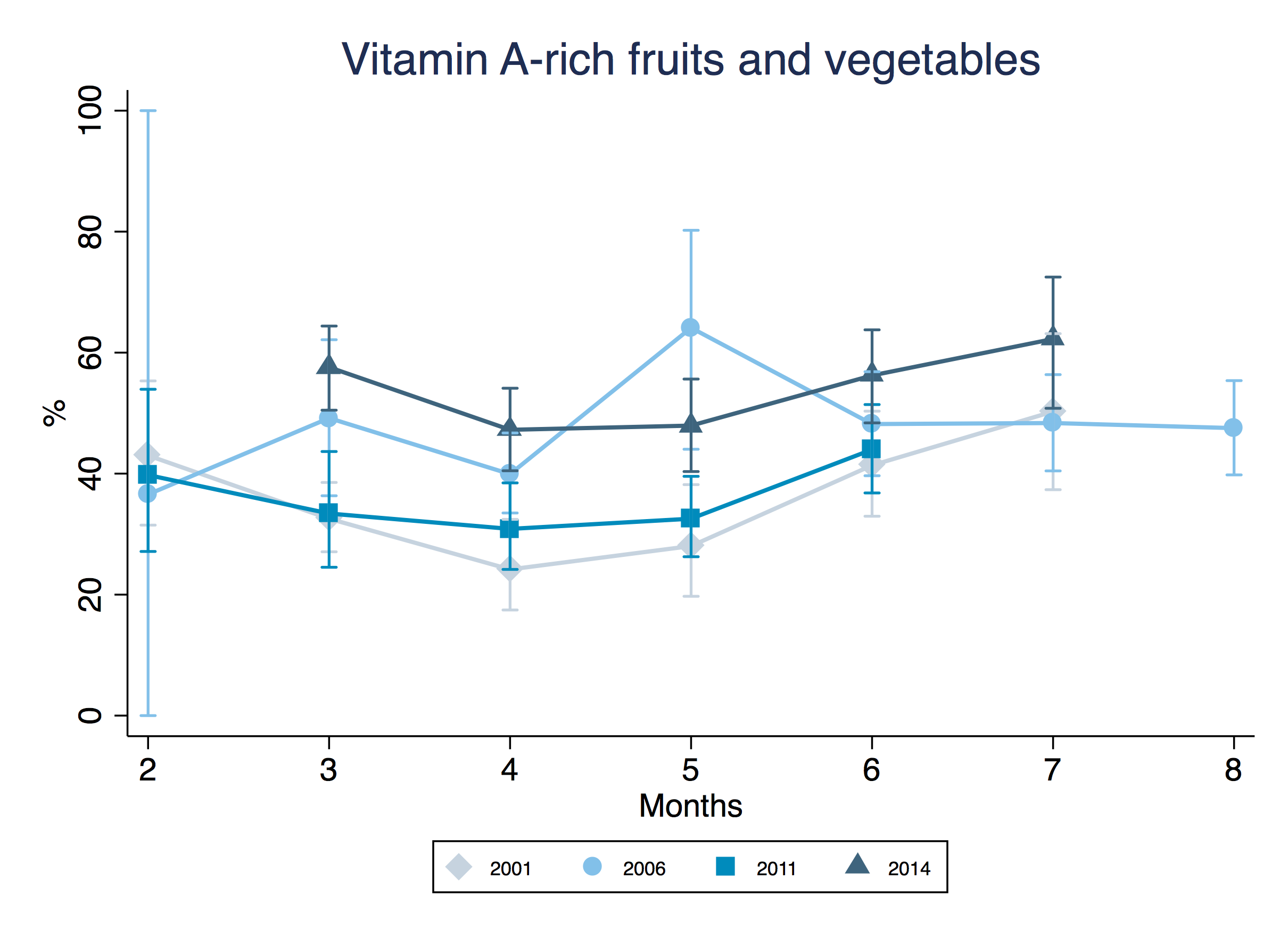


**Supplemental Figure 5:** Proportion of children consumed flesh foods and eggs between 2006 to 2014 by child age


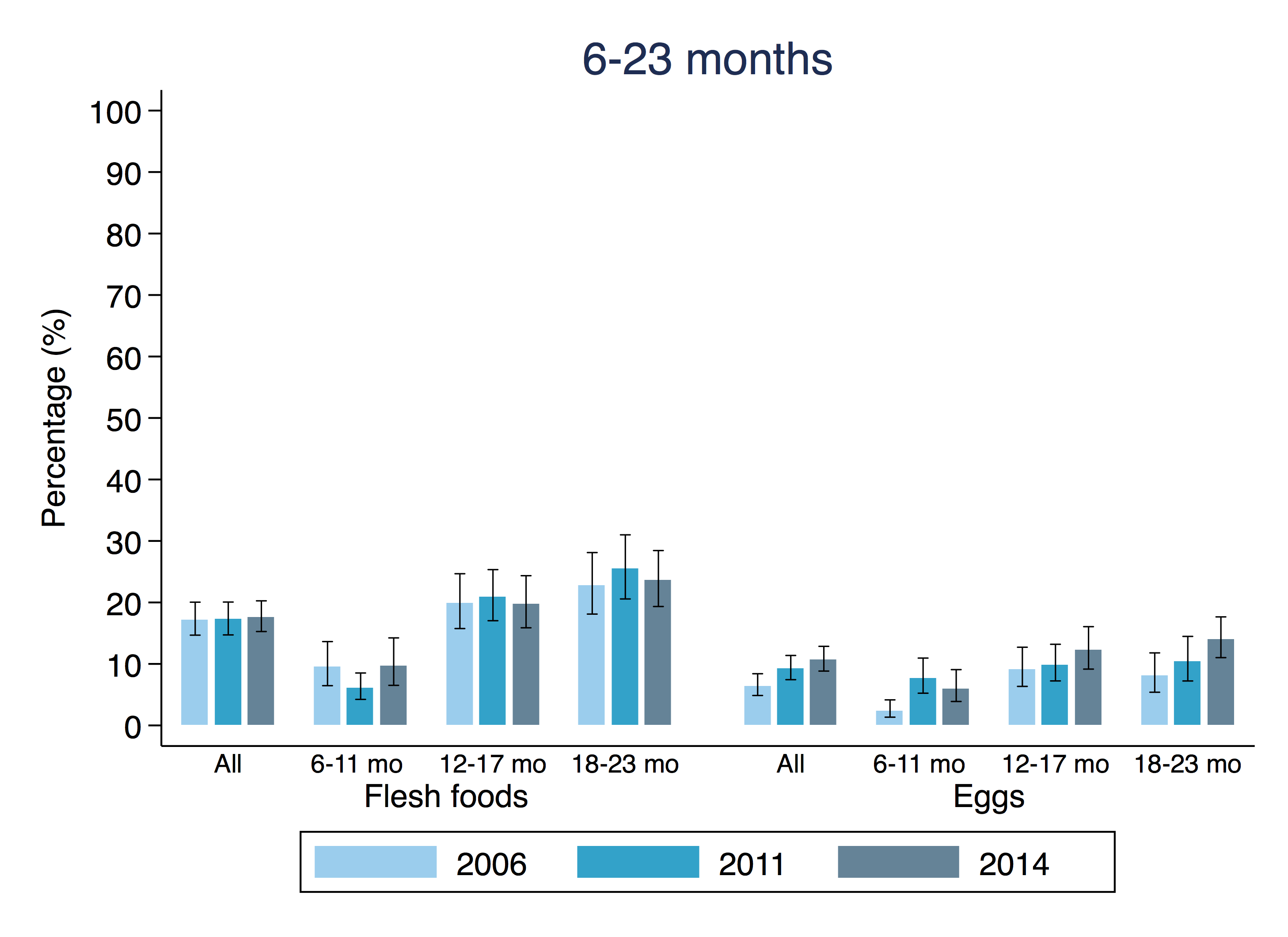

Supplement: Supplementary file 1 — Figure S1: Estimated proportion of appropriate complementary feeding practices over time by child sex. Pairwise slope comparison by delta methods: **, p < 0.01. Figure S2: Estimated proportion of appropriate complementary feeding practices over time by child age. Pairwise slope comparison by delta methods: **, p < 0.01. Figure S3: Estimated proportion of appropriate complementary feeding practices over time by maternal age. Pairwise slope comparison by delta methods: *, p < 0.05; **, p < 0.01. Figure S4: Proportion of vitamin A‐rich fruits and vegetables intake by year and by months of survey Figure S5: Proportion of children consumed flesh foods and eggs between 2006 to 2014 by child age [file MCN-14-e12564-s001.docx]
